# Genome-Wide Integration Site Profiling of Multi-Component CAR T-Cell Engineering Systems with ADA1/CD26 Co-Transduction

**DOI:** 10.3390/biomedicines14091941

**Published:** 2026-08-29

**Authors:** Kevin Song, Yue Hu, Xiaotong Song

**Affiliations:** 1 Department of Biology, University of Houston, Houston, TX 77004, USA; 2 Department of Translational Medical Sciences, Texas A&M University, Houston, TX 77030, USA; 3 Center for Inflammatory and Infectious Diseases, Institute of Biosciences and Technology, Texas A&M University, Houston, TX 77030, USA

**Keywords:** CAR T-cell, retroviral vector, viral integration, genome-wide profiling

## Abstract

**Background**: Retroviral vectors remain widely used for CAR T-cell manufacturing, including emerging multi-component engineering strategies that incorporate additional functional modules to enhance therapeutic performance. However, genome-wide profiling of integration patterns in CAR T cells subjected to multiplex retroviral engineering has not previously been reported. **Methods**: We performed genome-wide profiling of retroviral integration sites in CAR T-cell products derived from two targeting platforms (IL15-GPC3 and HER2). Each platform was evaluated under conditions with or without co-transduction of a retroviral vector encoding ADA1 and CD26. Experiments were conducted using cells obtained from a single donor with three technical replicates. Vector copy number (VCN), chromosomal distribution, genomic feature annotation, and integration hotspot analyses were conducted. **Results**: VCN levels were comparable across all CAR T-cell products and were not significantly affected by ADA1/CD26 co-expression. Integration events were broadly distributed across the genome with enrichment in gene-dense chromosomal regions. Chromosome 19 showed a prominent integration preference, with a secondary enrichment observed on chromosome 17. The majority of integration sites were located within intronic regions, with no enrichment observed in promoter regions. Recurrent integration hotspots were identified; however, no evidence of dominant clonal expansion was observed across conditions. Comparative analyses between ADA1/CD26 and control groups showed minimal differences in integration patterns. **Conclusions**: To our knowledge, this study provides the first comprehensive, genome-wide characterization of retroviral integration site profiles in CAR T-cell products manufactured by multi-vector co-transduction with an ADA1/CD26 module. By employing harmonized comparison with external reference datasets, we offer a directly benchmarked integration site characterization of multi-component CAR T-cell manufacturing. These findings establish a reference framework for integration site monitoring in next-generation CAR T-cell engineering systems and inform risk evaluation for emerging combinatorial vector strategies.

## 1. Introduction

Retroviral vectors are widely used for the genetic engineering of chimeric antigen receptor (CAR) T cells due to their stable genomic integration and sustained transgene expression [1,2,3,4,5,6,7,8,9,10]. Despite their clinical success, integration of retroviral vectors into the host genome remains an important consideration for insertional mutagenesis, clonal expansion, and overall product safety [2,11,12,13,14]. Previous studies have demonstrated that retroviral integration is non-random and preferentially occurs within gene-dense and transcriptionally active regions of the genome [15,16].

Comprehensive profiling of viral integration sites has therefore become an important component of CAR T-cell product characterization. Prior analyses, largely focused on single-vector CAR T-cell systems, have consistently demonstrated enrichment of integration events within intronic regions and gene-rich chromosomes, with limited evidence of promoter-associated oncogenic activation in currently used clinical platforms. However, CAR T-cell engineering strategies are becoming increasingly sophisticated, and the genomic integration behavior of multi-component engineering approaches remains less well characterized.

Recent advances in CAR T-cell design have incorporated additional functional modules intended to improve persistence, metabolic fitness, and antitumor activity within the tumor microenvironment. These include cytokine support systems such as IL15, as well as metabolic modulators including adenosine deaminase 1 (ADA1) and CD26 [17,18,19,20,21,22,23,24]. Notably, co-transduction using separate retroviral vectors has already been implemented in clinically relevant CAR T-cell platforms [19,25]. For example, co-transduction of IL15 and GPC3-CAR retroviral vectors has advanced to clinical evaluation in the United States [19]. However, despite the increasing translational and clinical use of retroviral co-transduction approaches, the genome-wide integration characteristics associated with multi-component CAR T-cell engineering have not been systematically characterized.

Given the potential implications for genomic stability, vector copy number, and clonal architecture, understanding how multi-vector engineering influences retroviral integration patterns is important for the development of next-generation CAR T-cell therapies. Whether the addition of auxiliary functional modules alters chromosomal distribution, genomic targeting preference, or hotspot formation compared with conventional single-vector systems remains unclear.

In this study, we perform a genome-wide characterization of retroviral integration sites in CAR T-cell products generated using IL15-GPC3 and HER2 targeting platforms, with or without ADA1/CD26 co-transduction. Our analysis focuses on vector copy number, integration distribution, and genomic feature annotation, in a multi-vector manufacturing context [15]. This dataset is intended to support manufacturing-level characterization of increasingly complex CAR T-cell engineering strategies.

## 2. Materials and Methods

### 2.1. CAR T-Cell Generation and Retroviral Transduction

Human peripheral blood mononuclear cells (PBMCs) obtained from healthy donors were purchased from STEMCELL Technologies (Cambridge, MA, USA). T cells were activated using anti-CD3/CD28 stimulation in the presence of recombinant human IL-2 under standard culture conditions.

CAR T cells were generated using γ-retroviral vectors encoding either IL15-GPC3 or HER2 CAR constructs. For multi-component engineering, activated T cells were additionally co-transduced with a second retroviral vector encoding ADA1 and CD26 (MR). Transductions were performed using retronectin-assisted retroviral transduction according to established protocols [20,21]. Transduced cells were expanded in culture for downstream analyses.

Transduction efficiency was assessed by flow cytometry based on CAR expression and/or transgene-associated marker expression, as described in our previous studies. CAR T-cell products analyzed in this study included IL15-GPC3, IL15-GPC3-MR, HER2, and HER2-MR groups. NT and MR serve as control groups. The MR-only group serves as a control to evaluate whether co-transduction with a CAR vector influences integration characteristics of the MR vector.

### 2.2. Vector Copy Number (VCN) Analysis

Vector copy number (VCN) per cell was quantified by droplet digital PCR (ddPCR) using vector-specific primer and probe sets targeting retroviral transgene sequences. Genomic DNA extracted from CAR T-cell products was analyzed using duplex ddPCR reactions containing both vector-specific and single-copy reference gene assays. Primer and probe sequences used for ddPCR analysis are listed in Table 1.

Droplet generation, PCR amplification, and droplet reading were performed according to the manufacturer’s recommended protocols. Absolute copy numbers for vector-derived and reference gene targets were calculated based on positive droplet counts using Poisson distribution analysis. VCN values were determined by normalizing vector copy numbers to the single-copy genomic reference gene and are reported as the average number of integrated vector copies per diploid genome.

### 2.3. Genomic DNA Extraction and Integration Site Library Preparation

Genomic DNA was isolated from CAR T-cell products using standard genomic DNA purification methods according to the manufacturer’s instructions. Viral integration site libraries were prepared using ligation-mediated PCR (LM-PCR) amplification of vector–genome junction fragments.

Briefly, genomic DNA was fragmented, ligated to adaptor sequences, and amplified using primers specific for retroviral long terminal repeat (LTR) sequences and adaptor regions. Amplified integration junction libraries were purified and subjected to high-throughput sequencing. Sequencing was performed to identify chimeric reads spanning vector and human genomic sequences for downstream integration site mapping analysis.

### 2.4. Sequencing Quality Control and Integration Site Mapping

Raw paired-end sequencing reads were adapter- and quality-trimmed using Trim Galore! (v0.6.10) with default parameters, including removal of Illumina adapter sequences and trimming of low-quality bases based on Phred quality scores [12,26,27,28,29]. Per-sample sequencing quality metrics were summarized using MultiQC (v1.30) to confirm overall read quality [29,30,31,32,33,34,35].

Viral integration site analysis was performed using the nf-core/viralintegration workflow with default parameters [36]. Reads were aligned to the human reference genome (*GRCh38*, Ensembl release 115) together with a viral reference composed of the integrating CAR T-cell vector constructs.

Intermediate chimeric read-calling outputs generated prior to the internal validation step of the pipeline were exported and subjected to custom filtering procedures. Chimeric reads were retained only if at least 20 bp aligned to the human genome and at least 20 bp aligned to the viral reference sequence. In addition, reads were retained only when the alignment score of the chimeric configuration was closer to the theoretical maximum alignment score than to the corresponding non-chimeric alignment score. Reads failing any of these criteria were excluded from downstream analyses (Table 2 and Table 3). For integration-site calling, the human genomic breakpoint coordinate was defined for each retained chimeric read and used for subsequent integration site analyses.

1. The number of reads passing filtering where more than or equal to 20 bp had to have aligned to the Human reference.
2. The number of reads passing filtering where more than or equal to 20 bp had to have aligned to the viral reference.
3. The number of reads passing filtering where the alignment score attributed to the read more closely matched a chimeric read alignment score than a non-chimeric read score.

### 2.5. Fuzz Collapsing of Integration Sites

To account for differences in chromosome length, minor mapping variability and local clustering of integration events, breakpoint positions were fuzz-collapsed by merging integration sites located within a ±5 bp genomic window on the same chromosome and treating them as a single integration event. We report the number of fuzz-collapsed unique integration sites per megabase (Mb) for each chromosome. Unless otherwise indicated, unique integration sites reported in this study refer to these fuzz-collapsed loci.

Fuzz collapsing was performed to minimize redundancy arising from closely spaced insertion events and potential PCR amplification artifacts while preserving broader integration distribution patterns and hotspot identification (Table 4).

### 2.6. Genomic Annotation of Integration Sites

Genomic annotation of viral integration sites was performed in R (v4.5.1) using the ChIPseeker and GenomicFeatures packages with a GTF-based gene annotation model for the human reference genome [37,38].

Integration loci were annotated relative to genomic features including promoter-associated regions, exons, introns, untranslated regions (UTRs), and intergenic regions. Promoter regions were defined as 2 kb upstream to 500 bp downstream of transcription start sites (TSS). Annotations corresponding to 5′ UTR and 3′ UTR were combined into a unified UTR category, while exonic and intronic annotations were grouped into exon and intron categories, respectively.

For each sample, counts and proportions of integration events within each genomic feature category were calculated.

### 2.7. Integration Hotspot and Differential Integration Analysis

For each sample, unique integration loci were summarized at the gene level by counting the number of integration events mapping to individual annotated genes. Integration hotspot analysis was performed to identify recurrently targeted genomic loci across CAR T-cell products. For visualization, hotspot genes were defined as genes with integration events detected in more than one technical replicate. Because all libraries were generated from the same donor, these represent technical rather than biological recurrence.

Differential integration analysis was performed by comparing integration frequencies between IL15-GPC3 and IL15-GPC3-MR CAR T-cell products, as well as between HER2 and HER2-MR CAR T-cell products. Fisher’s exact test was used to identify genes with differential enrichment of viral integration between groups.

Log2 fold changes were calculated as the log2 ratio of the proportion of total integration events occurring within a given gene in MR-containing CAR T-cell products relative to corresponding non-MR controls. Resulting *p*-values were adjusted for multiple testing using the Benjamini–Hochberg (BH) correction method. Genes with adjusted *p*-values ≤ 0.05 and absolute log2 fold change > 1 were considered significantly differentially integrated. Clonal abundance was determined by counting sequencing reads mapping to each unique, fuzz-collapsed integration site. A clone was classified as “dominant” if a single integration site accounted for >10% of all integration site–specific reads in a given sample, following established conventions. A clone was classified as “dominant” if a single integration site accounted for >10% of all integration site-specific reads within a sample. This threshold was used as a predefined operational criterion to identify relatively expanded clones for comparative comparisons and should not be interpreted as evidence of abnormal clonal expansion or insertional mutagenesis. Only one time-point (post-manufacture) was analyzed per product. No technical or biological replicates were extended to later culture periods or in vivo environments in this study.

### 2.8. Statistical Analysis

All downstream data processing, integration-site summarization, and visualization were performed in R (v4.5.1). Data are presented as mean ± standard deviation (SD) unless otherwise indicated. Statistical analyses were performed using GraphPad Prism (Version 11) and/or R statistical software (v4.5.1). To quantitatively compare genome-wide chromosomal integration patterns between groups, chromosome-level integration densities (unique integration sites per megabase, sites/Mb) were compared using a chi-square goodness-of-fit test. For each comparison, chromosome-specific integration densities were normalized to the total integration density across all chromosomes to generate relative chromosomal distributions. The distributions of HER2-CAR and HER2-MRCAR, IL15-GPC3-CAR and IL15-GPC3-MRCAR were then compared. A two-sided *p* < 0.05 was considered statistically significant. Other comparisons between groups were performed using appropriate statistical tests as indicated in the figure legends. A *p* value < 0.05 was considered statistically significant.

## 3. Results

### 3.1. Vector Copy Number Across CAR T-Cell Products

**Figure 1:**
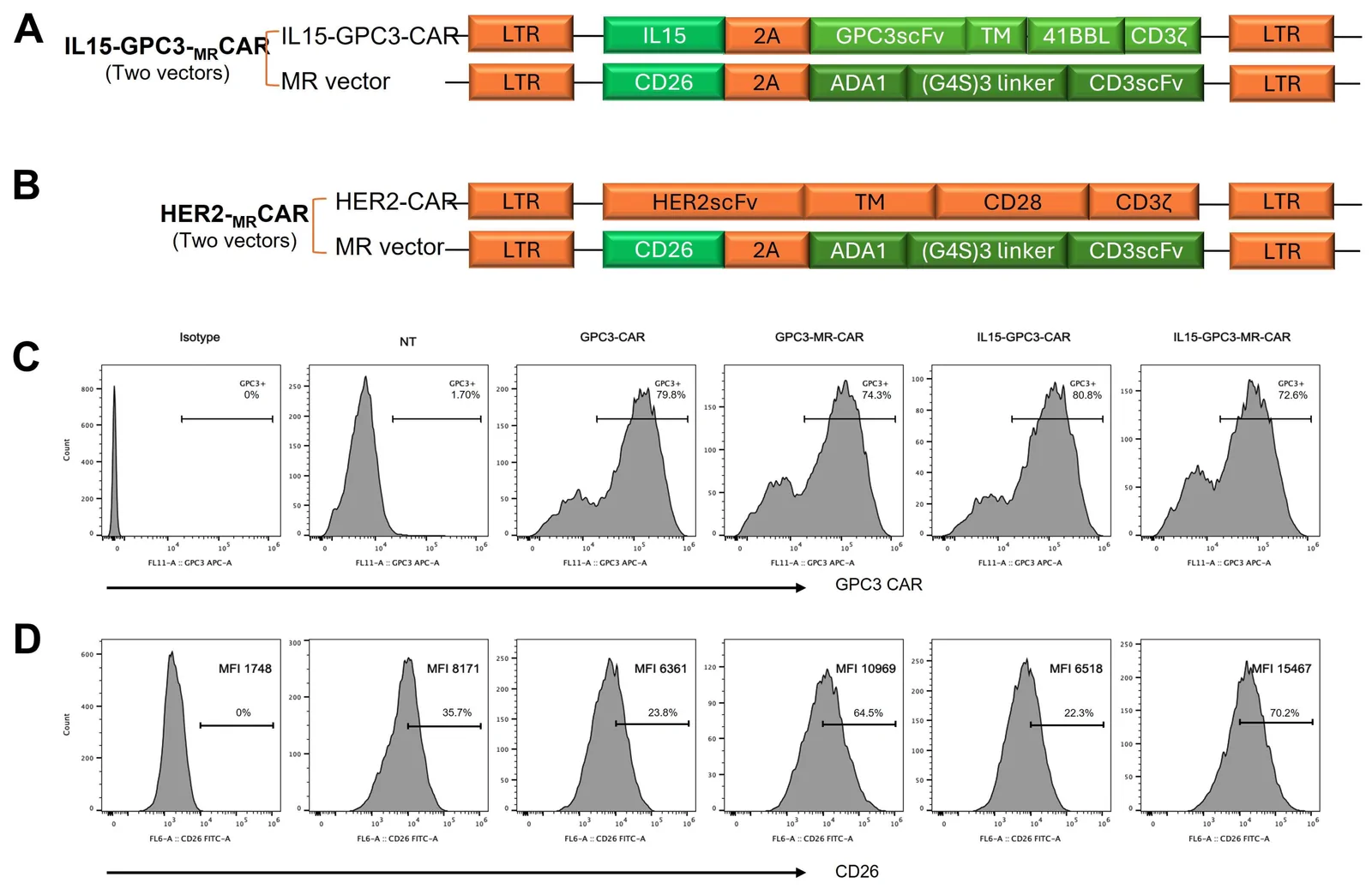

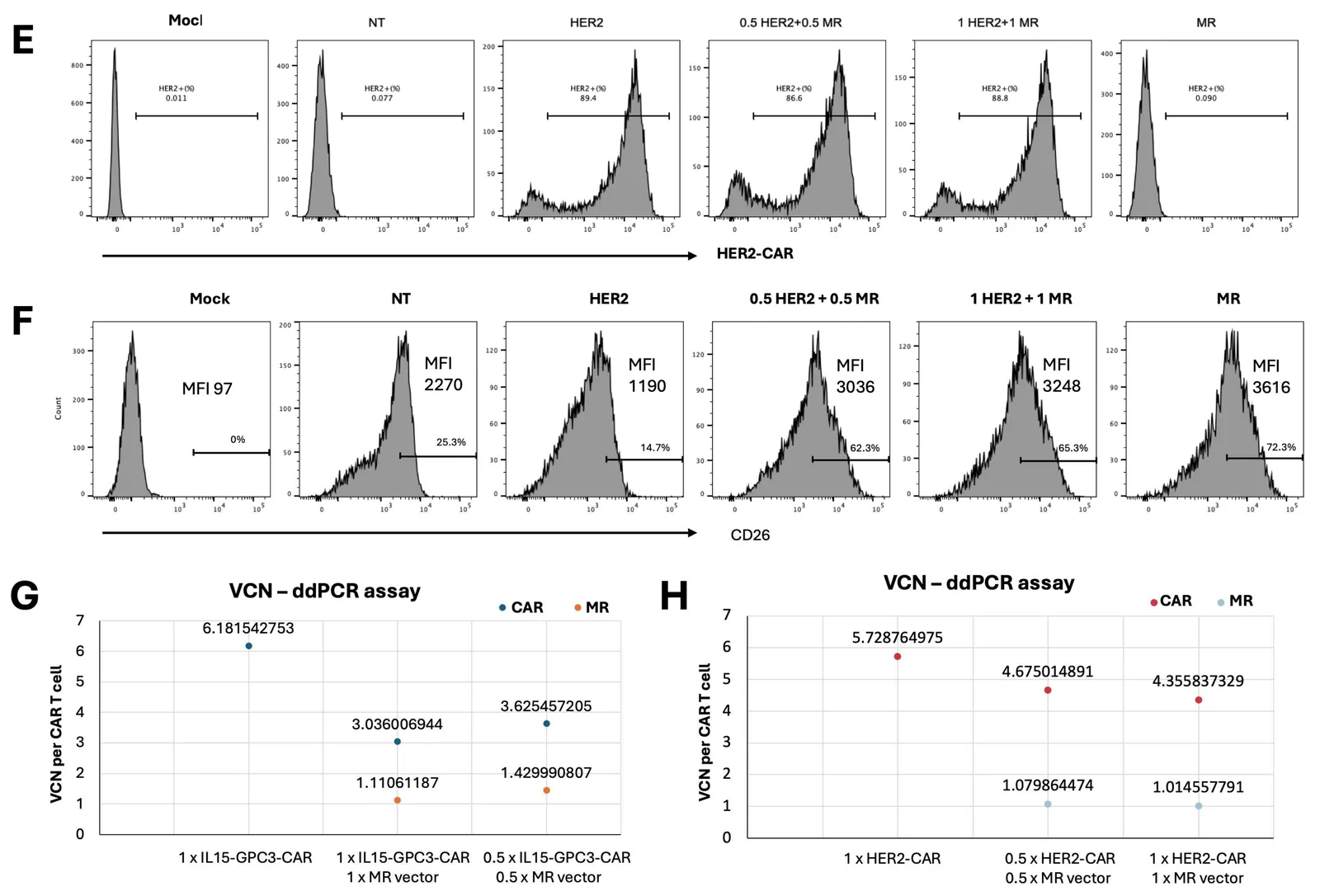
Engineering strategy, transduction efficiency, and vector copy number analysis of multi-component CAR T-cell products. (**A**) Schematic representation of the IL15-GPC3-MR CAR T-cell engineering strategy. CAR T cells were generated using a two-vector retroviral transduction system consisting of an IL15-GPC3-CAR vector encoding IL15-2A-GPC3scFv-TM-4-1BB-CD3ζ and a separate MR retroviral vector encoding CD26 and ADA1-CD3scFv separated by a 2A peptide sequence. (**B**) Schematic representation of the HER2-MR CAR T-cell engineering strategy. HER2 CAR T cells were generated using a retroviral vector encoding HER2scFv-TM-CD28-CD3ζ together with co-transduction of the MR retroviral vector encoding CD26 and ADA1-CD3scFv separated by a 2A peptide sequence. (**C**) Representative flow cytometry analysis of GPC3-CAR expression in IL15-GPC3 and IL15-GPC3-MR CAR T-cell products following retroviral transduction. NT represents no transduction with CAR or MR vectors, while GPC3-CAR contains the GPC3-targeting CAR alone; GPC3-MRCAR co-expresses the GPC3 CAR with the ADA1/CD26 metabolic regulator (MR); IL15-GPC3-CAR co-expresses the GPC3 CAR with IL-15; and IL15-GPC3-MRCAR co-expresses the GPC3 CAR with both IL-15 and the ADA1/CD26 metabolic regulator. (**D**) Representative flow cytometry analysis of CD26 expression in IL15-GPC3-MR CAR T-cell products. Quantitative flow cytometric analysis showed that the percentages of CAR/CD26 double-positive cells were 18.99%, 48.315%, 18.02%, and 50.97% for the GPC3-CAR, GPC3-MR-CAR, IL15-GPC3-CAR, and IL15-GPC3-MR-CAR groups, respectively. (**E**) Representative flow cytometry analysis of HER2-CAR expression in HER2 and HER2-MR CAR T-cell products following retroviral transduction. (**F**) Representative flow cytometry analysis of CD26 expression in HER2-MR CAR T-cell products. The percentages of CAR/CD26 double-positive cells were 13.14%, 53.95%, and 57.99% for the HER2-CAR, 0.5 HER2/0.5 MR, and 1 HER2/1 MR groups, respectively. Cell viability and expansion were comparable among all groups, with no statistically significant differences observed. (**G**) Vector copy number (VCN) analysis of IL15-GPC3 and IL15-GPC3-MR CAR T-cell products determined by droplet digital PCR (ddPCR) using vector-specific primers. (**H**) Vector copy number (VCN) analysis of HER2 and HER2-MR CAR T-cell products determined by ddPCR. VCN was determined by ddPCR using pooled genomic DNA from each manufactured CAR T-cell product. Comparable VCN levels were observed between MR-containing and control CAR T-cell products.

We first evaluated vector copy number (VCN) across CAR T-cell products generated from IL15-GPC3 and HER2 targeting platforms, with or without co-transduction of a retroviral vector encoding ADA1/CD26 (MR). All experiments were performed using samples derived from a single donor with three technical replicates. Therefore, the analyses evaluate technical reproducibility across experimental conditions within a single donor and should not be interpreted as evidence of biological variability or consistency across multiple donors. VCN analysis demonstrated comparable levels of vector integration across all groups (Figure 1), indicating that incorporation of the MR construct did not substantially increase overall vector burden within engineered T cells.

Although minor variability in VCN was observed between individual products, no consistent increase was detected in MR-containing CAR T cells relative to their corresponding parental controls. These findings suggest that dual-vector transduction incorporating ADA1/CD26 can be achieved without excessive accumulation of integrated vector copies. Importantly, the observed VCN ranges remained within levels commonly reported for retroviral CAR T-cell manufacturing platforms, supporting the feasibility of multi-component engineering strategies using conventional retroviral transduction approaches.

### 3.2. Genome-Wide Distribution of Viral Integration Sites

**Figure 2:**
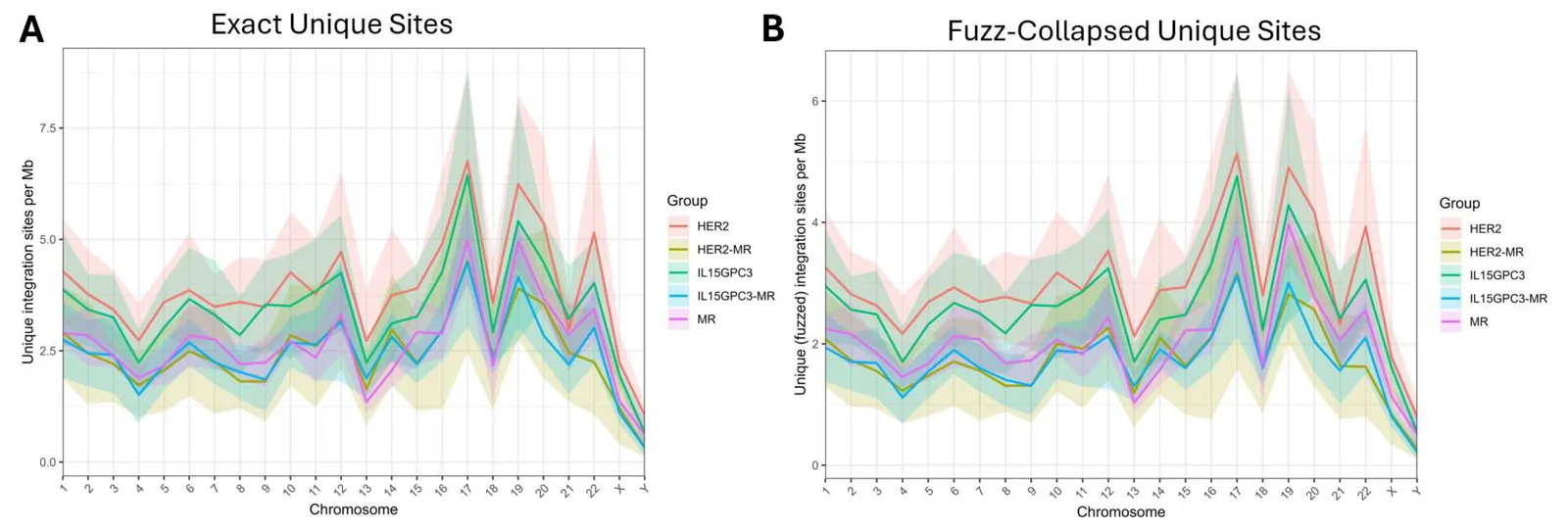
Genome-wide chromosomal distribution of viral integration sites in CAR T-cell products. Unique viral integration site density is shown across human chromosomes for IL15-GPC3, IL15-GPC3-MR, HER2, and HER2-MR CAR T-cell groups. For each group, the lines represent the mean number of (**A**) exact and (**B**) fuzz collapsed unique integration sites per megabase (sites/Mb) on each chromosome, averaged across three libraries per group. Shaded ribbons indicate ±1 standard deviation from the group mean, reflecting between-library variability. Chi-square test was used to compare the genome-wide chromosomal distributions. No significant differences were observed between HER2 and HER2-MRCAR products (χ^2^ = 1.78, df = 23, *p* > 0.999) or between IL15-GPC3 and IL15-GPC3-MRCAR products (χ^2^ = 2.94, df = 23, *p* > 0.999). Statistical comparisons were performed on integration-site distributions. Because the three libraries per group represent technical replicates rather than independent biological donors, these analyses should be interpreted as descriptive support for distributional similarity rather than formal inference regarding biological variability. Integration events were broadly distributed across the genome, with preferential enrichment in gene-dense chromosomes. Chromosome 19 demonstrated a prominent integration peak, and a secondary enrichment was observed on chromosome 17.

To characterize the global integration landscape of engineered CAR T cells, we analyzed the chromosomal distribution of unique viral integration sites across all products. Integration events were broadly distributed throughout the genome and were detected across nearly all chromosomes (Figure 2), consistent with previously reported retroviral integration patterns [15]. Integration density was enriched in gene-dense chromosomes, particularly chromosomes 17 and 19. Chromosome 19 demonstrated a prominent peak in integration frequency that was highly consistent with published reference datasets. In contrast, our dataset additionally revealed a secondary enrichment on chromosome 17, which was less pronounced in the reference profile.

After normalization for chromosome length using fuzz-collapsed unique integration sites per Mb, chromosomes 19 and 17 continued to demonstrate higher integration site densities relative to other chromosomes. This enrichment is not attributable to differences in chromosome size, but rather indicates a genuine preference of the retroviral vectors, consistent with previous reports highlighting the targeting of gene-dense genomic regions. We note that other genomic features—such as gene density, transcriptional activity, chromatin accessibility, and sequence mappability—can affect both true biological integration preferences and technical detectability. Although not quantitatively adjusted for in this analysis, these influences are likely reflected in the observed integration patterns.

Both chromosomes 17 and 19 are among the most transcriptionally active and gene-rich regions of the human genome, suggesting that chromatin accessibility and transcriptional status continue to influence retroviral integration preferences in these CAR T-cell products. The additional chromosome 17 enrichment observed in our dataset may reflect biological variability associated with donor material, CAR construct composition, or sequencing depth.

### 3.3. Distribution of Integration Sites Across Genomic Features

**Figure 3:**
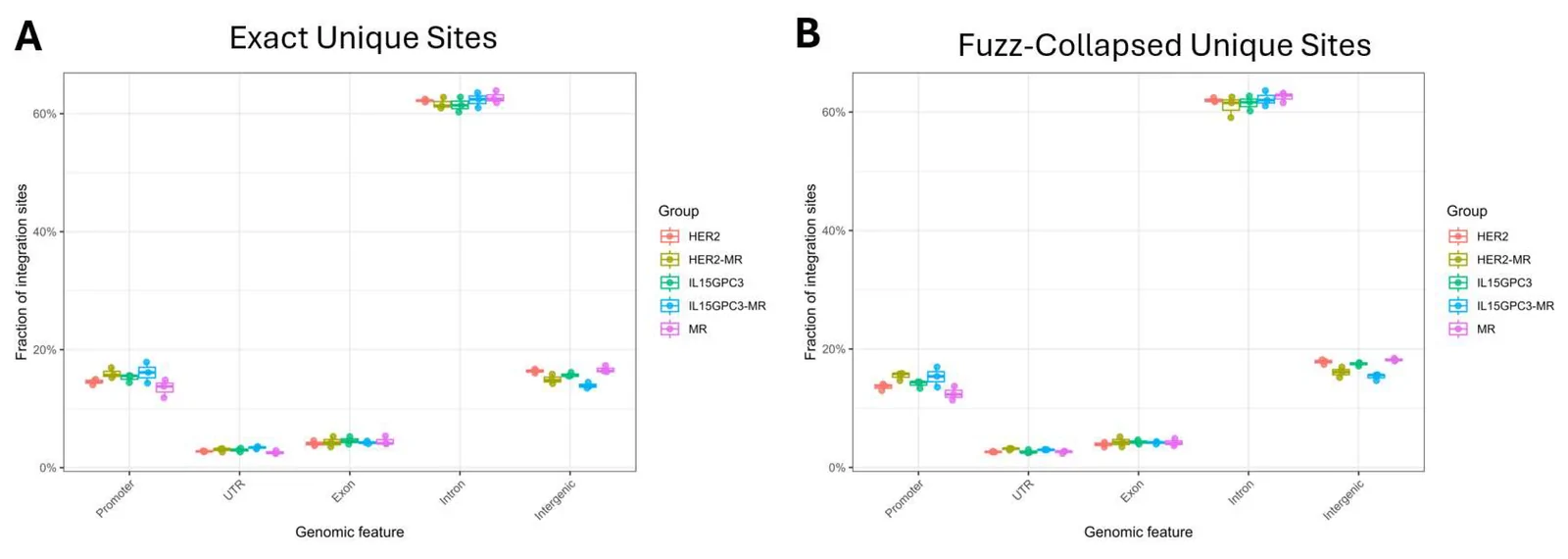
Distribution of integration sites across genomic features. The genomic annotation of viral integration sites is shown for IL15-GPC3, IL15-GPC3-MR, HER2, and HER2-MR CAR T-cell products. Boxplots depict the fraction of unique integration sites that localize to promoter, UTR, exon, intron, or intergenic regions, stratified by construct group, for (**A**) exact and (**B**) fuzz collapsed integration sites. Individual points represent per-sample fractions, computed by mapping integration sites to the GRCh38 gene model (Ensembl v115) and annotating features using ChIPseeker, with promoters defined as 2 kb upstream to 500 bp downstream of the transcription start site. The y-axis reflects the proportion of integration sites within each feature (number of sites in a feature divided by the total integration sites for that sample). Genomic feature annotation distributions were compared using a chi-square test. No significant differences were observed between HER2-CAR and HER2-MRCAR products (χ^2^ = 0.020, df = 4, *p* > 0.99) or between IL15-GPC3-CAR and IL15-GPC3-MRCAR products (χ^2^ = 0.018, df = 4, *p* > 0.99), indicating that ADA1/CD26 co-transduction did not alter the overall genomic feature preferences of retroviral integration.

We next investigated the genomic context of viral integration events by annotating integration sites relative to genomic features, including introns, exons, promoter regions, untranslated regions (UTRs), and intergenic regions (Figure 3). Across all CAR T-cell products, integration events were predominantly located within intronic regions, representing the major class of detected insertion events. Lower frequencies of integration were observed within exonic regions, promoter-associated regions, and untranslated regions. Intergenic integration events were also detected but occurred at lower frequencies relative to intronic insertions.

The predominance of intronic integration supports the conclusion that integration targeting in these CAR T-cell products follows established genomic preferences. Importantly, no enrichment of integration events within promoter regions was observed in MR-containing CAR T-cell products compared with their corresponding controls.

Comparison between IL15-GPC3 and HER2 platforms revealed highly similar genomic feature distributions, suggesting that CAR target specificity and ADA1/CD26 co-expression do not substantially influence preferential integration into particular genomic compartments. Collectively, these findings indicate that the addition of ADA1/CD26 modules does not shift retroviral integration toward regulatory genomic elements associated with increased genotoxic risk.

### 3.4. Hotspots of Viral Integration at Gene Loci

**Figure 4:**
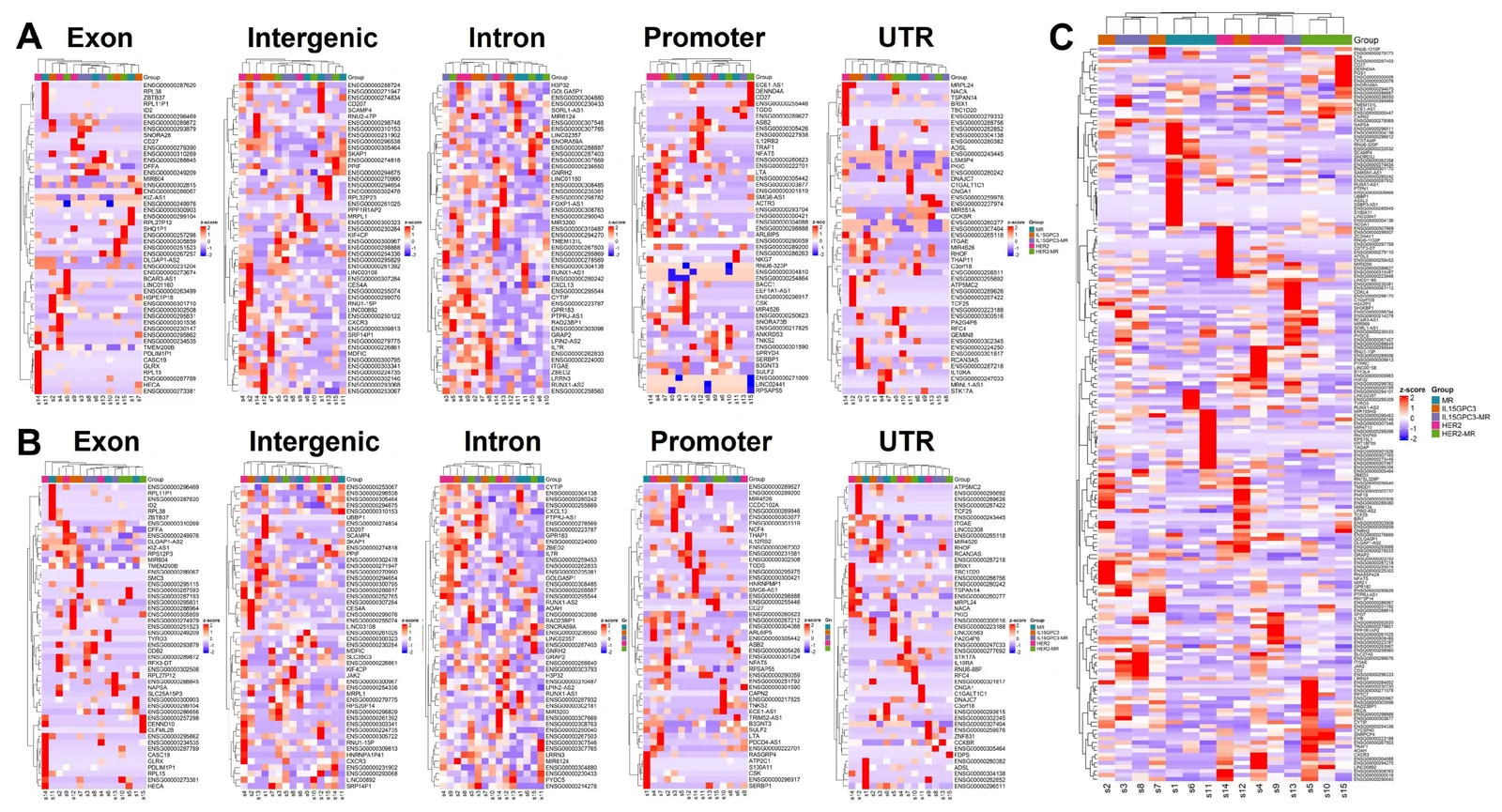
Hotspots of viral integration at gene loci. (**A**) Recurrently targeted genomic loci identified using unique viral integration sites across CAR T-cell products. Integration hotspots were defined based on recurrent vector insertion events detected within the same gene loci across samples. (**B**) Integration hotspot analysis following fuzz-collapsing of nearby integration events within a defined genomic window to reduce redundancy arising from local clustering and PCR amplification bias. Fuzz-collapsing reduced apparent redundancy while preserving the overall hotspot distribution pattern. Recurrent integration events were distributed across multiple loci without evidence of dominant clonal expansion. Similar hotspot patterns were observed across IL15-GPC3, IL15-GPC3-MR, HER2, and HER2-MR CAR T-cell products. (**C**) Top 50 genes across each product are presented.

To further assess integration site clustering and potential clonal expansion patterns, we performed hotspot analysis at the gene level using both unique integration sites and fuzz-collapsed integration sites (Figure 4). Analysis of unique integration sites identified several recurrently targeted loci across samples. These recurrent events likely reflect preferential integration into transcriptionally active genomic regions rather than selective clonal expansion, as integration hotspots were distributed across multiple independent loci rather than concentrated within a limited number of dominant genes.

To minimize potential amplification bias arising from closely spaced integration events, we additionally applied fuzz-collapsing analysis, in which neighboring insertion events within a defined genomic window were merged into single integration events. Following fuzz-collapsing, the overall hotspot distribution remained largely preserved, although the apparent frequency of certain clustered loci was reduced.

Importantly, no individual hotspot demonstrated overwhelming dominance following fuzz-collapsing analysis, arguing against the presence of strong clonal outgrowth driven by vector integration. Similar hotspot distributions were observed across all CAR T-cell products, including MR-containing constructs, further supporting the stability of integration behavior following multi-component retroviral engineering.

### 3.5. Differential Integration Patterns Between CAR T-Cell Products

**Figure 5:**
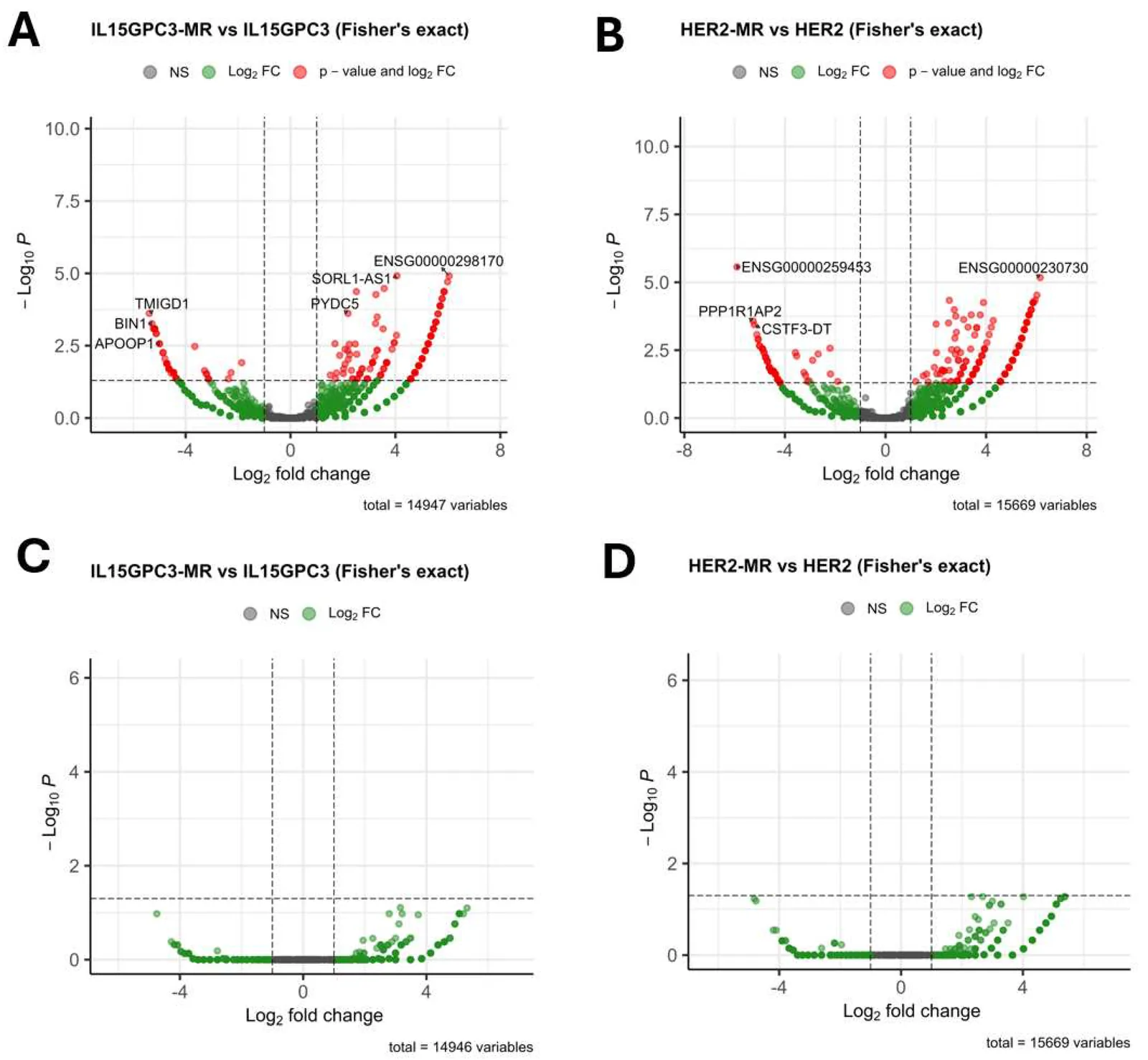
Differential integration analysis between CAR T-cell products. Differential integration site analysis comparing IL15-GPC3 versus IL15-GPC3-MR CAR T-cell products and HER2 versus HER2-MR CAR T-cell products. Differential integration frequencies were calculated at the gene-locus level using both unique integration sites (**A**,**B**) and fuzz-collapsed integration datasets (**C**,**D**). Only modest differences in integration frequencies were observed between MR-containing and non-MR CAR T-cell products. Many apparent differences identified using unique integration sites were reduced following fuzz-collapsing analysis, suggesting that local clustering effects contributed to the initial differential signals. Overall, the global integration landscapes remained highly similar across all CAR T-cell products, indicating that ADA1/CD26 co-expression did not substantially alter retroviral integration targeting behavior.

To determine whether ADA1/CD26 co-expression influenced integration site selection, we performed differential integration analysis comparing IL15-GPC3 versus IL15-GPC3-MR and HER2 versus HER2-MR CAR T-cell products (Figure 5). Comparative analysis identified modest differences in integration frequencies at selected genomic loci between MR-containing and non-MR products. However, these differences were generally limited in magnitude and were not associated with consistent enrichment of specific genomic pathways or chromosomal regions.

Notably, many of the initially identified differential integration events were reduced following fuzz-collapsing analysis, suggesting that local clustering of closely spaced insertion events contributed to apparent differences detected in the uncollapsed dataset. After normalization using fuzz-collapsed integration sites, the overall integration landscapes between MR-containing and control CAR T-cell products remained highly similar. These findings indicate that incorporation of ADA1/CD26 modules through dual retroviral transduction does not substantially alter global integration site selection or clonal architecture within engineered CAR T-cell products. Collectively, the data support the genomic stability and reproducibility of multi-component CAR T-cell engineering approaches.

## 4. Discussion

In this study, we performed genome-wide profiling of retroviral integration sites across CAR T-cell products generated from IL15-GPC3 and HER2 targeting platforms, with or without co-expression of ADA1/CD26 modules. Our results demonstrate that integration patterns in these multi-component CAR T-cell systems remain broadly consistent with established retroviral integration behavior and are not substantially altered by incorporation of additional functional modules.

Retroviral integration site profiling has become increasingly important in the development of engineered cell therapies, particularly as CAR T-cell platforms continue to evolve toward more complex multi-component systems. While stable genomic integration is a major advantage of retroviral vectors for durable transgene expression, integration into the host genome also raises concerns regarding insertional mutagenesis, clonal dominance, and long-term genomic stability. Most prior studies evaluating integration patterns in CAR T cells have focused on single-vector systems, whereas relatively limited information is available regarding integration behavior in dual-vector or multifunctional engineering strategies.

Consistent with previous reports, we observed preferential integration in gene-dense chromosomes, particularly chromosome 19. This preferential integration pattern likely reflects the tendency of retroviral vectors to target transcriptionally active chromatin regions. In addition to chromosome 19 enrichment, our dataset demonstrated a secondary peak on chromosome 17. Because both chromosomes are characterized by high gene density and transcriptional activity, this observation may reflect a combination of chromatin accessibility and dataset-specific sampling variability rather than a distinct shift in integration preference. Differences in sequencing depth between datasets may also contribute to variation in observed integration frequencies.

Analysis of genomic feature distribution demonstrated that integration events occurred predominantly within intronic regions, while promoter-associated integrations remained relatively infrequent across all CAR T-cell products. This pattern is highly consistent with previously described retroviral integration profiles and does not suggest preferential targeting of regulatory regions associated with increased genotoxic risk. Importantly, incorporation of ADA1/CD26 modules did not increase promoter-associated integration events, supporting the genomic stability of the multi-component engineering strategy used in this study.

Our hotspot analysis identified recurrently targeted loci across samples; however, these events were distributed across multiple genomic regions and did not demonstrate evidence of dominant clonal expansion at the analyzed manufacturing time point. This observation is important because selective outgrowth of clones harboring integration events near growth-promoting genes has historically represented a major safety concern in integrating vector systems. The absence of strong clonal dominance in our dataset suggests that the observed recurrent integration events primarily reflect known retroviral preferences for accessible chromatin rather than integration-driven selective expansion.

We additionally performed fuzz-collapsing analysis to reduce redundancy arising from closely spaced insertion events and local amplification artifacts. Following fuzz-collapsing, many apparent differential integration events between MR-containing and control CAR T-cell products were substantially reduced. The disappearance of statistically significant gene associations after fuzz-collapsing likely reflects multiple factors. While fuzz-collapsing is intended to reduce inflation of enrichment signals caused by local clustering of nearby integration sites, it also reduces the number of independent integration events available for analysis, thereby decreasing statistical power. Consequently, the loss of significance should not be interpreted solely as evidence that the original signals were artifacts. Several genes identified in the uncollapsed analysis, including *TMIGD1, BIN1, SORL1-AS1*, and *PPP1R1AP2*, did not remain significant after fuzz-collapsing. Although some of these genes have reported roles in cellular processes such as cell adhesion, endocytosis, or neurological function, none are established recurrent insertional oncogenesis targets in hematopoietic cells. Furthermore, the lack of significance after correction suggests that these associations are not sufficiently robust to support a biologically meaningful enrichment.

To further evaluate the safety profile of the vector system, we compared the recurrently targeted genes identified in this study with genes previously implicated in insertional mutagenesis in retroviral and lentiviral gene therapy, including *LMO2, MECOM* (EVI1), *PRDM16,* and *CCND2*. None of these established common insertion site genes showed recurrent enrichment or differential targeting in our dataset. Moreover, the most frequently targeted genes (Figure 4) did not overlap substantially with genes associated with insertional oncogenesis. Although our study is limited by the use of a single donor and technical replicates, these findings provide no evidence of preferential integration into genomic loci previously linked to clonal expansion or malignant transformation. Overall, our findings indicate that there is no consistent evidence for preferential integration near genes associated with clonal selection or transformation, supporting the safety profile of the vector system evaluated in this study.

The present study has several limitations. First, the analysis was performed using pre-infusion CAR T-cell products and therefore does not evaluate long-term in vivo clonal dynamics following adoptive transfer. Second, we did not directly adjust for gene density, transcriptional activity, or chromatin accessibility in our normalization algorithm. Future studies employing integrative approaches with chromatin profiling or transcriptome data may yield further insight into the specificity of vector integration patterns. Third, while integration site analysis provides important information regarding genomic distribution patterns, it does not directly assess functional transcriptional consequences associated with individual insertion events. Future studies integrating transcriptomic analysis, longitudinal clonal tracking, and in vivo persistence data may further clarify the biological impact of integration events in multifunctional CAR T-cell platforms. Next, statistical comparisons were performed on integration-site distributions. Because the three libraries per group represent technical replicates rather than independent biological donors, these analyses should be interpreted as descriptive support for distributional similarity rather than formal inference regarding biological variability. An additional limitation is that hotspot and clonal dominance analyses were performed using three technical replicate libraries derived from a single donor. Consequently, the observed hotspot patterns cannot be interpreted as evidence of recurrent integration across independent biological samples. Furthermore, clonal abundance was estimated from raw chimeric read counts without unique molecular identifiers (UMIs), and PCR amplification bias may have influenced the apparent abundance of individual integration sites. Future studies including multiple donors, biological replicates, longitudinal sampling, and UMI-based library preparation will provide a more robust assessment of integration hotspots and clonal dynamics. Last, although we did not observe dominant clonal expansion at the manufacturing endpoint, our analysis was limited to a single time point. Future studies incorporating extended in vitro culture and/or longitudinal in vivo tracking are warranted to fully assess the risk of post-manufacturing clonal dominance.

Despite these limitations, our findings provide important technical and translational insights into the genomic behavior of multi-component CAR T-cell engineering approaches. As next-generation CAR T-cell therapies increasingly incorporate cytokine support systems, metabolic regulators, synthetic signaling modules, and combinatorial transgene strategies, evaluation of integration site distribution and clonal architecture will remain an important component of product characterization and safety assessment.

In conclusion, our data demonstrate that co-expression of ADA1/CD26 in IL15-GPC3 and HER2 CAR T-cell platforms does not substantially alter retroviral integration behavior, genomic feature targeting, or clonal architecture at the analyzed manufacturing time point. These findings support the genomic stability of multi-component CAR T-cell engineering approaches and provide a practical framework for evaluating integration patterns in increasingly complex next-generation cell therapy products.

## Figures and Tables

**Table 1:** Primers and probes used for ddPCR-based vector copy number (VCN) analysis. This table summarizes the primer and probe sequences used for droplet digital PCR (ddPCR)-based quantification of vector copy number (VCN) in CAR T-cell products. Vector-specific primer/probe sets targeting retroviral transgene sequences were used to detect integrated CAR vector copies, while a single-copy human reference gene was included for genomic normalization. ADA1 and CD26 are expressed from the same bicistronic vector separated by a 2A peptide, allowing quantification using a single primer/probe set VCN values were calculated as the ratio of vector-derived copies to reference gene copies per diploid genome.

Primer/Probe	Sequence
*ATP2B4* FWD	ACT CAG TGC GCA AGT CAA T
*ATP2B4* REV	GCG CAA GAT GAT CTC AGA GG
*ATP2B4* PRB	/5HEX/TT GGG ATT C/ZEN/C TGA TGA CGG TGC TC/3IABkFQ/
HER2CAR FWD	GAT TGG ATA TGG AGG ATC CTG TTC
HER2CAR REV	AGT CTC TCC GGG CTT CTT
HER2CAR PRB	/56-FAM/TC ACA GCG A/ZEN/A GTC CAG CTG CA/3IABkFQ/
*GPC3* FWD	TCA GGA GCC GAA CTG GT
*GPC3* REV	GTG CAT CTC GTA GTC TGT GAA G
GPC3 PRB	/56-FAM/TG CAT GAC A/ZEN/G TTT GAC ACT TGC GC/3IABkFQ/
*ADA1*/CD26 FWD	GTC AAG TTC AAC CGC CTA CA
*ADA1*/CD26 REV	CCA GGC AGT AAT GGT CAT CAT AG
*ADA1*/CD26 PRB	/56-FAM/TG CGC AGT A/ZEN/G TAC ACG GCA GAA TC/3IABkFQ/

**Table 2:** Average number of chimeric reads identified and retained after filtering. Chimeric reads were identified using the nf-core/viralIntegration workflow and subjected to a series of filtering steps. This table displays the average number of ‘raw’ (unfiltered) chimeric reads and the average number of reads retained after the filtering step for each CAR T-cell product group. Detailed sample-level values for each filtering step are provided in the following slide.

Group	Average # Raw Reads	Average # Filtered Reads
MR	672,845	607,200
IL15GPC3	625,780	561,279
IL15GPC3-MR	1,143,919	1,097,832
HER2	653,834	584,045
HER2-MR	1,032,776	989,729

**Table 3:** Number of chimeric reads per sample at each filtering step. This table shows the number of chimeric reads identified for each sample at each stage of the filtering process using the nf-core/viralIntegration workflow. Values are reported for each sample and each filtering step, including both unfiltered (raw) reads and reads retained after filtering. Samples 01–03 represent technical replicates.

Sample ID	Raw # Chimeric Reads	Reads Passing: 20 bp Human (1)	Reads Passing: 20 bp Viral (2)	Reads Passing: Chimeric Alignment Score (3)
Sample1 (01_MR)	781,839	724,864	712,222	707,353
Sample2 (01_ILG)	689,137	641,104	627,761	623,400
Sample3 (01_ILG_MR)	1,157,321	1,121,061	1,115,743	1,111,136
Sample4 (01_H)	708,057	662,019	651,637	645,771
Sample5 (01_H_MR)	1,244,417	1,206,382	1,200,881	1,197,359
Sample6 (02_MR)	625,504	589,942	575,271	570,813
Sample7 (02_ILG)	613,606	570,613	560,295	555,486
Sample8 (02_ILG_MR)	1,012,129	982,664	978,066	975,210
Sample9 (02_H)	553,473	512,677	505,621	500,138
Sample10 (02_H_MR)	1,070,865	1,039,006	1,033,758	1,031,172
Sample11 (03_MR)	611,192	560,006	550,051	543,434
Sample12 (03_ILG)	574,598	521,039	512,112	504,952
Sample13 (03_ILG_MR)	1,262,307	1,218,364	1,212,196	1,207,150
Sample14 (03_H)	699,972	629,038	615,802	606,227
Sample15 (03_H_MR)	783,048	749,447	743,955	740,656

**Table 4:** Number of unique viral integration sites identified per sample, before and after fuzz-collapsing. Viral integration sites were called at single-base pair resolution and reported as unique integration sites. To account for minor mapping jitter, a “fuzz-collapsed” dataset was also generated by merging sites within ±5 bp on the same locus into a single event. This table shows the number of exact and fuzz-collapsed integration sites for each sample following filtering. While fuzz-collapsing alters the absolute counts, it does not affect the overall pattern of integration across chromosomes. Accordingly, downstream figures are presented with both exact and fuzz-collapsed site versions. Samples 01–03 represent technical replicates.

Sample ID	Unique Integration Sites	Fuzz Collapsed Integration Sites
Sample1 (01_MR)	8871	5688
Sample2 (01_ILG)	13,760	8731
Sample3 (01_ILG_MR)	9491	5365
Sample4 (01_H)	15,541	9906
Sample5 (01_H_MR)	10,896	6235
Sample6 (02_MR)	8321	5070
Sample7 (02_ILG)	9868	6153
Sample8 (02_ILG_MR)	7035	3827
Sample9 (02_H)	10,149	6363
Sample10 (02_H_MR)	6606	3570
Sample11 (03_MR)	6563	4309
Sample12 (03_ILG)	7578	4971
Sample13 (03_ILG_MR)	5616	3240
Sample14 (03_H)	9356	6078
Sample15 (03_H_MR)	4636	2594

## Data Availability

The data presented in this study are openly available in Gene Expression Omnibus (GEO) at https://www.ncbi.nlm.nih.gov/geo/, accessed on 30 June 2026.

## Abbreviations

The following abbreviations are used in this manuscript:

CAR	Chimeric antigen receptor
HER2	Human epidermal growth factor receptor
GPC3	Glypican 3
ADA1	Adenosine deaminase 1
MR	Metabolic refueling
GO	Gene ontology
KEGG	Kyoto Encyclopedia of Genes and Genomes
